# Common pre-diagnostic features in individuals with different rare diseases represent a key for diagnostic support with computerized pattern recognition?

**DOI:** 10.1371/journal.pone.0222637

**Published:** 2019-10-10

**Authors:** Lorenz Grigull, Sandra Mehmecke, Ann-Katrin Rother, Susanne Blöß, Christian Klemann, Ulrike Schumacher, Urs Mücke, Xiaowei Kortum, Werner Lechner, Frank Klawonn

**Affiliations:** 1 Department of Pediatric Hematology and Oncology, Hannover Medical School, Hannover, Germany; 2 Nursing Council (Pflegekammer) Lower Saxony, Hannover, Germany; 3 Department of Pediatrics and Adolescent Medicine, University of Cologne, Cologne, Germany; 4 Department of Hematology, Hemostasis, Oncology and Stem Cell Transplantation, Hannover Medical School, Hannover, Germany; 5 Department of Pediatric Pneumology, Allergy and Neonatology, Hannover Medical School, Hannover, Germany; 6 DRK Clementinenkrankenhaus, Hannover, Germany; 7 Department of Computer Science, Ostfalia University of Applied Sciences, Wolfenbuettel, Germany; 8 Improved Medical Diagnostics IMD GmbH, Donauwoerth, Germany; 9 Biostatistics, Helmholtz Centre for Infection Research, Braunschweig, Germany; Politechnika Krakowska im Tadeusza Kosciuszki, POLAND

## Abstract

**Background:**

Rare diseases (RD) result in a wide variety of clinical presentations, and this creates a significant diagnostic challenge for health care professionals. We hypothesized that there exist a set of consistent and shared phenomena among all individuals affected by (different) RD during the time before diagnosis is established.

**Objective:**

We aimed to identify commonalities between different RD and developed a machine learning diagnostic support tool for RD.

**Methods:**

20 interviews with affected individuals with different RD, focusing on the time period before their diagnosis, were performed and qualitatively analyzed. Out of these pre-diagnostic experiences, we distilled key phenomena and created a questionnaire which was then distributed among individuals with the established diagnosis of i.) RD, ii.) other common non-rare diseases (NRO) iii.) common chronic diseases (CD), iv.), or psychosomatic/somatoform disorders (PSY). Finally, four combined single machine learning methods and a fusion algorithm were used to distinguish the different answer patterns of the questionnaires.

**Results:**

The questionnaire contained 53 questions. A total sum of 1763 questionnaires (758 RD, 149 CD, 48 PSY, 200 NRO, 34 healthy individuals and 574 not evaluable questionnaires) were collected. Based on 3 independent data sets the 10-fold stratified cross-validation method for the answer-pattern recognition resulted in sensitivity values of 88.9% to detect the answer pattern of a RD, 86.6% for NRO, 87.7% for CD and 84.2% for PSY.

**Conclusion:**

Despite the great diversity in presentation and pathogenesis of each RD, patients with RD share surprisingly similar pre-diagnosis experiences. Our questionnaire and data-mining based approach successfully detected unique patterns in groups of individuals affected by a broad range of different rare diseases. Therefore, these results indicate distinct patterns that may be used for diagnostic support in RD.

## Introduction

Collectively, rare diseases are not rare. By definition, a rare or orphan disease is one that affects fewer than 1:5000 individuals. Today, there are more than 7000 different known RD, affecting an estimated of 350 million people worldwide [1]. However, because of the small patient populations for each individual RD, funding to investigate causes and treatments is limited, slowing the discovery of diagnostic tools and potential therapies [2]. This is one reason that diagnosing RD presents a complex clinical challenge. The length of time from onset of symptoms to an accurate diagnosis is about 5 years for RD. In a quarter of cases, the diagnostic delay ranges between 5 and 30 years [1, 3, 4]. The longer it takes to diagnose a RD, the more physicians the patient has seen. On average, RD patients see more than 7 different physicians before a diagnosis is made [1]. Reasons for this delay, sometimes called ‘diagnostic odyssey’ are multiple. Firstly, patients, their families, and the treating physicians, often have limited awareness of RD. In addition, the symptoms are often mingled with or mistaken for symptoms of more common diseases. Initially, these symptoms may be considered minor, and thus of little concern [4–7]. Lastly, symptoms of RD may not be evident to doctors or nurses who have never encountered the particular disease in question. These obstacles result in a significant burden for the affected patients, as well as for the health care system at large [8,9]. Delays in making the correct diagnosis may lead to inappropriate management as well as uncontrolled disease progression, sometimes resulting in irreversible sequelae. Misdiagnosis can also lead to unnecessary interventions at significant additional risk to patients [10–12]. Therefore, initiatives to improve and shorten pre-diagnostic time periods are needed and several programs addressing these issues in regard to RD have been launched by the European Union [13].

In pilot studies, we sought to develop a data-mining and questionnaire-based diagnostic support tool for selected rare diseases, including primary ciliary dyskinesia and glycogen storage disease type II (also known as Pompe disease). Using combined machine learning methods for questionnaires that were completed by patients, this pattern recognition system achieved an average sensitivity of 90% in pilot studies [14,15]. From these experiences, we learned that patients’ experiences during the diagnostic journey could be harnessed to create a support tool to aid physicians in the early diagnosis of a RD.

The current project proved the hypothesis that individuals with completely different RDs share similar pre-diagnostic experience. Several RD with the greatest need for diagnostic support were selected after a Delphi survey was conducted among experts in Germany [16]. We interviewed individuals with 21 different RDs and built a database of answered questionnaires. Combined machine learning methods were then employed to differentiate questionnaires of individuals with rare disease (RD), common non-rare disease (NRO), chronic diseases (CD) and individuals with psychosomatic disorders (PSY). The study goal was to develop a diagnostic support tool for individuals with an undiagnosed RD using a new questionnaire and machine learning classifier.

## Methods

### Delphi survey, interviews and machine learning methods

In this monocentric, prospective pilot study, we tested whether the subjective experiences and views of patients with selected RD could provide diagnostic support in most rare diseases with a long diagnostic latency. All patients or their legal guardians gave their informed consent for the interview. The study received ethics committee approval by the ethics committee of the Medical University of Hannover (no.: 2316–2014; head on time of approval: Prof. H.D. Tröger).

In order to identify those RD with the longest diagnostic latency periods and the most acute demand for diagnostic support, we previously performed and published a Delphi survey among German experts for RD [16].

Briefly, German experts on RD were contacted twice to name those RD, where diagnostic support is particularly needed. In order to cover a preferably broad spectrum of RD with the interviews, RD with different characteristics were selected (e.g. RD with visible signs as acromegaly versus invisible such as cluster headache or RD typically affecting children (mucopolysaccharidosis typ1 1) versus RD affecting adults, such as amyotrophic lateral sclerosis. Likewise, 21 RD were systematically selected for interviews. In the next step and to gain insight into patients’ view of the pre-diagnostic process, we conducted interviews with patients diagnosed with 21 patients or relatives of those RDs identified in the Delphi survey. Affected individuals or their parents were contacted through patient advocacy groups and invited to share their experiences during their pre-diagnostic journey. In total, 21 interviews were performed lasting between 63 and 450 minutes. Most interviews (19/21) were conducted in the family’s home. In 9/21 interviews, the parents participated in place of their minor children. The diseases included were amyotrophic lateral sclerosis, Ehlers-Danlos syndrome (EDS), congenital glaucoma, ornithine transcarbamylase (OTC) deficiency, McArdle disease, Pompe disease, cluster headache, Fanconi anemia, sclerodermia, acromegaly, Hurler syndrome, pulmonary arterial hypertension, Wilson disease, myelodysplatic syndrome, cystic fibrosis, severe combined immune-deficiency, ataxia teleangiectasia, periodic fever syndrome and Fabry disease. In addition, one interview was conducted with an individual with somatoform disorder and another with an individual whose diagnosis could be established despite an extensive search.

The interviews took place across Germany between February 2015 and May 2015 by four authors (SB, AR, US and LG). These semi-structured (narrative) interviews always started with the same initial question (“Would you please tell me everything that comes to mind from the time before your diagnosis was established. Please just tell me everything you consider to be of any importance and share your observations of all occurrences“). When the patient finished sharing their thoughts, the interviewer initiated further questioning to elucidate more details.

All interviews were digitally recorded, transcribed and analyzed according to the Colaizzi technique [17]. Consequently, an inductive system of categories was built, reflecting the pre-diagnostic phenomena gathered during the interview process which finally resulted in a questionnaire containing 53 items (S1 File).

#### Systematic analysis of the interviews

Four researchers (SB, SM, AR and LG) independently reviewed and subsequently discussed interviews. Using the techniques described by Colaizzi, the observations of the patients extracted from the interviews were then systematically categorized. A step-wise qualitative analysis was performed, including extraction of significant phrases, reduction of the phrases to their essential structures, generation of a question out of the essential structures and validation of questions through interviewees. To sort the observations and create a questionnaire that reflected the relevant experiences, we classified the content of the interviews into categories according to the strategies employed in previous studies in patients with pulmonary or neuromuscular diseases [14,15]. Out of those categories, questions were generated, resulting in a questionnaire that includes all categories (S1 File). Likewise, the questionnaire reflected personal observations and experiences in all categories and consequently covered all the pre-diagnostic phenomena experienced by the interviewees. In close dialogue with patient support groups, a maximum length of two pages, and a completion time of ten minutes, was defined for the questionnaire. The answers in the questionnaire were scaled from 1 (“completely false”) to 4 (“completely true“). The questions were reviewed by interviewees and other patients, and the feedback gathered was used to enhance the comprehensibility of the final questionnaire, which contained 53 questions. We designed two questionnaires, one for sick individuals still in the process of reaching a diagnosis and an equivalent questionnaire for the parents of a sick child. As an example, six questions of the adult version questionnaire are displayed in Table 1; the complete questionnaire is provided in the supplementary material (S2 File).

**Table 1:** Example of questions (selection of 6 out of 53 questions; for the complete questionnaire: S1 file).

Did you suspect for a period of time prior to your diagnosis that something was wrong with your health?
Do you deliberately avoid activities or tasks that make your symptoms obvious to others?
Is it difficult for you to describe your complaints / symptoms?
Do you notice any special tricks or techniques you have developed to compensate for symptom-related limitations in mastering everyday tasks?
Can you recall a situation when your symptoms caused you to feel threatened?
Did you attempt to research possible causes for the complaints / symptoms you were experiencing?

#### Collection of answered questionnaires

Patients with an established diagnosis of a RD were invited to answer the questionnaire. To facilitate answering, a web-based platform and a paper-based version were created for the participants. Individuals without a RD, but with a chronic disease or a psychological disorder were contacted through the various departments at Hannover University Hospital (MHH). To increase awareness of the campaign, we contacted patient advocacy groups and set up a Facebook^™^ page explaining the project, with a link to the web-based version of the questionnaire.

#### Machine learning techniques and data selection

A classification method and an adopted version of a fusion algorithm were employed as previously described [14,15]. In brief, the current study based upon previous approaches by using four different classifier methods and three independent data sets to arrive at the final diagnostic suggestion. The concept to define independent data sets for corresponding disease groups with similar symptoms was successfully applied to the analysis of genetic data sets of patients with rare diseases in [18]. We used the following classifiers: support vector machine (SVM), random forest (RF), logistic regression (LR) and linear discriminant analysis (LDA). Each of the 4 single classifiers calculates a diagnosis with a corresponding probability value. In many cases, the same diagnosis was chosen by the 4 classifiers and a clear vote was delivered. However, sometimes a ‘diagnostic parity’ occurs for the 4 binary classifiers. Therefore, a further SVM classifier was trained (‘super-fusion classifier‘) which takes into account the pre-calculated and numerically coded diagnoses and the corresponding probability values of the 4 classifiers. Finding the best classifier is a matter of debate; working on classifying systems in questionnaires we realized that no individual classifier algorithm works perfectly as stand-alone method for classification of comparable sets of data. By contrast, the best combination of the four classifiers used in this study worked equal or better than the performance of each single classifier (S3 File). Besides, it was not the purpose of the paper to evaluate the performance of different classifiers but to apply a method that had been established in similar applications [14,15] and works in comparable scenarios. Statistics about the comparison of the classifiers can be found in the supplementary material (S3–S6 Files) showing that the fusion classifier is better than single classifiers but only sometimes with a difference of statistical significance.

There are two different types of input vectors to classify. The classifiers for the data sets evaluate an input vector that includes the answer options of the questionnaire. The answer “no” is mapped for each question to a numeric value of 1 and the opposite answer “yes” to a value of 4. This input vector consists of 55 elements containing the 53 answers to the questions, the gender and the age of the patient.

The results gained by the four classifiers are collected to create an input vector for the fusion SVM classifier. This fusion input vector contains an index for each classified disease and its corresponding probability value. This leads to an input vector with 4x2 = 8 elements (four index values and four probability values).

The classifier parameters were set to default values. For the SVM kernel a 3-dimensional-polynominal function was selected.

The collection of answered questionnaires was gathered from different sources as it was necessary to reach individuals with RD. As a result, questionnaires of patients with confirmed, assumed or unknown diagnoses were collected. As it was the study goal to focus on RD and to distinguish RD from chronic and psychosomatic / somatoform diseases, at the end of the pre-evaluation and the consolidation process the training set of questionnaires consisted of 440 patients (or questionnaires) with a confirmed diagnosis. This training set was further subdivided into three data sets due to medical similarities of the diagnosis and the comments given by patients within the questionnaires.

The training process is based on k-fold stratified cross-validation and results are presented by sensitivity values, specificity values and confusion matrices for each of the 4 binary classifiers as well as the fusion classifier.

Following a stepwise classification process, a given questionnaire is categorized into a diagnostic group in accordance with the main study question (the identification of a RD). We followed an approach similar to that proposed by Tsalik [18], in which separate independent classifiers were constructed for pairs of classes or disease groups, instead of one classifier distinguishing between all four groups selected in this study. Because the selected groups of NRO and RD are rather heterogeneous, the full data set was scaled down to 3 pairs of independent data subsets. Each subset pair consists out of two alternative disease groups. All three subsets represent the collection of similar diseases from a clinical point of view that takes into account comparable and groupable diseases.

The training algorithm software applies R statistic libraries controlled within a Java coding. The locally calculated classifier results are stored in data arrays and in the R package format. All data are uploaded to a Linux root server system of a public internet provider. The PHP application software of this web server evaluates the R functions by a software “OpenCPU” server system. A public software access to the questionnaire is possible by the link “www.imdresearch.de/selten” and a password. The diagnostic evaluation of a given questionnaire and the display of the diagnostic suggestions are protected by further hashed passwords taking into account privacy statements.

## Results

### Creation of a novel questionnaire

According to the qualitative analysis, four thematic groups could be generated from the interview material: 1. ‘perceiving symptoms’; 2. ‘searching for a diagnosis’, 3. ‘achieving symptom-control’ and 4. ‘efforts to adapt in daily life’

In these four thematic main groups, 33 sub-categories were identified. For the selection of a set of questions that best reflected the experience of RD patients, four workshops were performed. The main objective for the selection of questions was that all thematic groups and interviews be covered. A second requirement for the questionnaire was that it includes all essential topics collected in the qualitative analysis of the interviews. Accordingly, the final questionnaire contained 53 questions (Table 1 and S1 File).

### Return rate of questionnaires and further analysis

In total, 1763 individuals answered the questionnaire, about ¾ using the web-based version. 608 out of the 1763 questionnaires were excluded from further analysis (questionnaires from healthy individuals, or from individuals with incomplete questionnaires or individuals with diseases outside the scope of the study). In total, 1155 questionnaires qualified for inclusion in machine learning operations and for further test runs (Table 2).

Among the 1155 questionnaires used for the analysis and training there were three larger groups of single diseases (sarcoidosis n = 144, PAH n = 50 and syringomyelia n = 44). The other questionnaires were summarized under the term ‘umbrella RD’, such as rare endocrinological diseases or disorders of the skin. Besides the collection of the 758 individuals with a RD, 149 individuals with a non-rare chronic disease and 48 patients with psychological disorders answered the questionnaire.

Table 2 lists the structure of all of the answered questionnaires in the data set.

**Table 2:** Return rates of questionnaires.

**Rare diseases (RD) group and assigned RD subgroups**	
Sarcoidosis	144
PAH	50
Syringomyelia	44
SLE	31
Rare endocrinological diseases^a^	36
Rare neuromuscular diseases^b^	90
Rare diseases of the skin^c^	68
Rare neurological diseases^d^	93
Rare pain syndromes^e^	22
Rare autoimmune diseases^f^	94
Rare metabolic diseases^g^	52
Rare pulmonary diseases^h^	34
**Sum of all RD**	**758**
**Questionnaires assigned to non-rare diseases**	
NRO^i^	200
CD^k^	149
PSY	48
**Sum of all non-rare diseases**	**397**
**Questionnaires excluded**	
no diagnosis (online questionnaires)	349
incomplete questionnaires	225
healthy individuals	34
**Sum of all questionnaires not included in further analysis**	**608**
**Total sum of questionnaires for machine learning evaluation** **758 of rare + 397 of none-rare disease = 1155 questionnaires**	**1155**
**Total sum of all received questionnaires** **1155 used + 608 excluded questionnaires = 1763 questionnaires**	**1763**

^a^ Including patients with acromegaly, addisons disease, adenoma, cushings disease

^b^ Including patients with ALS, CIDP (chronic inflammatory demyelinating polyneuropathy), muscular dystrophy Duchenne, FSHD, SMA, PNP

^c^ Including patients with EDS, ectodermal dysplasia, epidermolysis bullosa, lipoedema, mastocytosis

^d^ Including patients with GBS, M. Menière, Arnold chiari malformation

^e^ Mostly patients with cluster headache

^f^ Including patients with M. Still, M. Wegener, M. Behcet, dermatomyositis, Moya-Moya syndrome

^g^ Including patients with Glycogenosis 1 to 9, M. Fabry, metachromatic leukodystrophia, Niemann-Pick Type C

^h^ Including mostly patients with PCD and Cystic Fibrosis

^I^ Patients feeling ill but without a conclusive diagnosis despite intensive workup. The rare disease center Bonn added 34 questionnaires from individuals without diagnosis despite intensive testing and searching

^k^ Including patients with asthma, inflammatory bowel disease

### Retrospective testing

The set of 1155 evaluable questionnaires were then separated into the 4 pre-defined groups of ‘RD, ‘NRO’, ‘CD’ and ‘PSY’. In a second step, pairs of diagnostic challenges were constructed (Table 3). Each single questionnaire belonged to only one of the data sets and each subset of the selected diseases was evaluated to allow separation between two main diagnostic groups. Table 3 documents the properties of the selected data subsets, which resulted from an intensive data analysis process. Those 715 questionnaires not used for training were utilized for prospective data evaluation.

**Table 3:** Structures of the 3 data subsets.

Data set	Classifier class 1	Classifier class 2	Questionnaires^c^
1	pulmonary hypertension (PAH), cystic fibrosis	other non-rare diseases (including patients w/o diagnosis)	90 : 90 → 180
2	Sarcoidosis, syringomyelia SLE^a^, acromegaly, Ehlers-Danlos-Syndromes; Morbus Still, Nail-Patella-Syndrom	chronic diseases	90 : 90 → 180
3	CIDP^b^, cluster headache, Ménièr‘s disease, Fabry disease	psychosomatic disorders	42 : 38 → 80

^a^ Systemic lupus erythematodes

^b^ Chronic inflammatory demyelinating polyneuropathy

^c^ A selection of questionnaires was chosen at random

**Fig 1:**
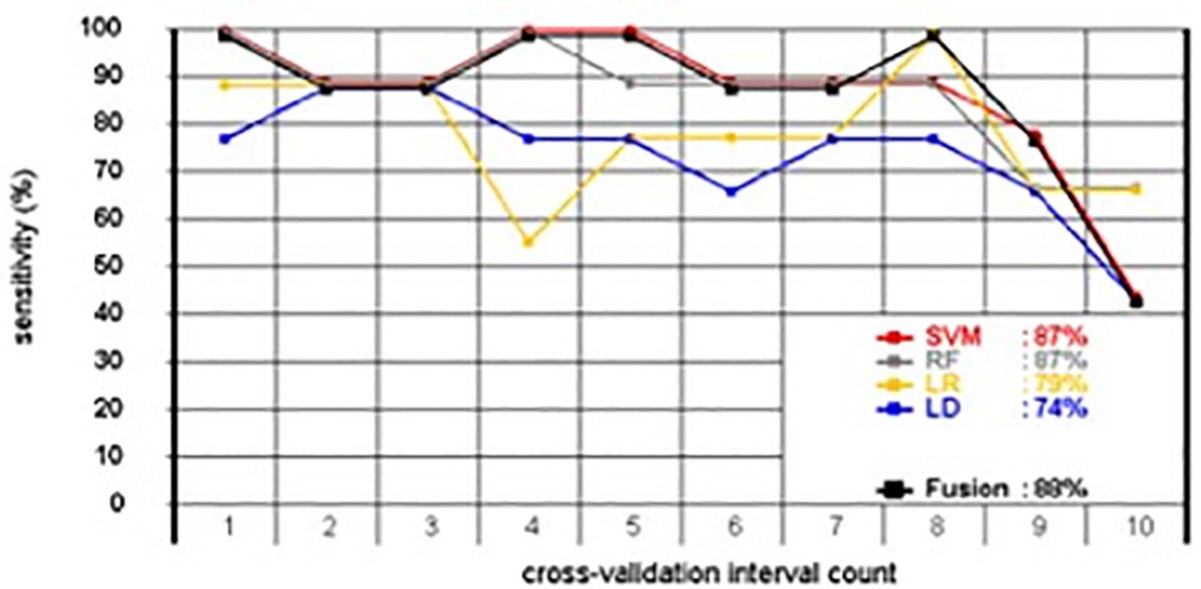
Sensitivity values of a 10-fold stratified cross-validation run. Data set 1 (RD versus NRO). The single diagnosis of the four different classifiers and their corresponding probabilities were evaluated by a further classifier, which computed the final diagnosis. For the fusion, a support vector machine (SVM, black line) was selected, because it performed best. For a better reading of the curves are shifted vertically with a few pixels.

For each single classifier (SVM, RF, LR, LD) and for each pair of the three data sets (Table 3) a 10-fold stratified cross-validation analysis was performed. A representative computer cross-validation run is displayed for data set 1 (‘RD versus ‘NRO’ diseases) in Fig 1. The sensitivity values of the fusion classifier are higher than the values of the single classifiers, indicating that the combination of different classifiers outperforms any single classifier in this setting (Fig 1).

In Table 4, the results of all stratified 10-fold cross-validation runs for the three data sets are displayed. The sensitivity values vary from 84.2% for the ‘PSY’ up to 93.3% for ‘RD’ in data set 2. Combining the three different sensitivity values for the rare diseases in the three independent data sets by a final aggregation algorithm reaches a sensitivity value for the detection of a RD with a mean value of 88.9%.

**Table 4:** Results of stratified 10-fold cross-validation runs for data set 1, 2 and 3. A binary confusion matrix is based on the results of cross-validation by counting the numbers of true positives (TP), false negatives (FN), false positives (FP) and true negatives. For data set 1 the TP values are assigned to the RD and the TN to the NRO. The TN numbers of data set 2 corresponds to the CD and the TN number of data set 3 to the PSY. The sensitivity values for all 3 data sets are defined by TP/(TP+FN) and the corresponding specificity is given by TN/(TN+FP).

Data set	Diagnostic groups	Sensitivity	Specificity	Confusion matrix
1	RD versus NRO	RD 87.7%	NRO 86.6%	79 TP / 11 FN / 12 FP / 78 TN
2	RD versus CD	RD 93.3%	CD 87.7%	84 TP / 6 FN / 11 FP / 79 TN
3	RD versus PSY	RD 85.7%	PSY 84.2%	36 TP / 6 FN / 6 FP / 32 TN

**Fig 2:**
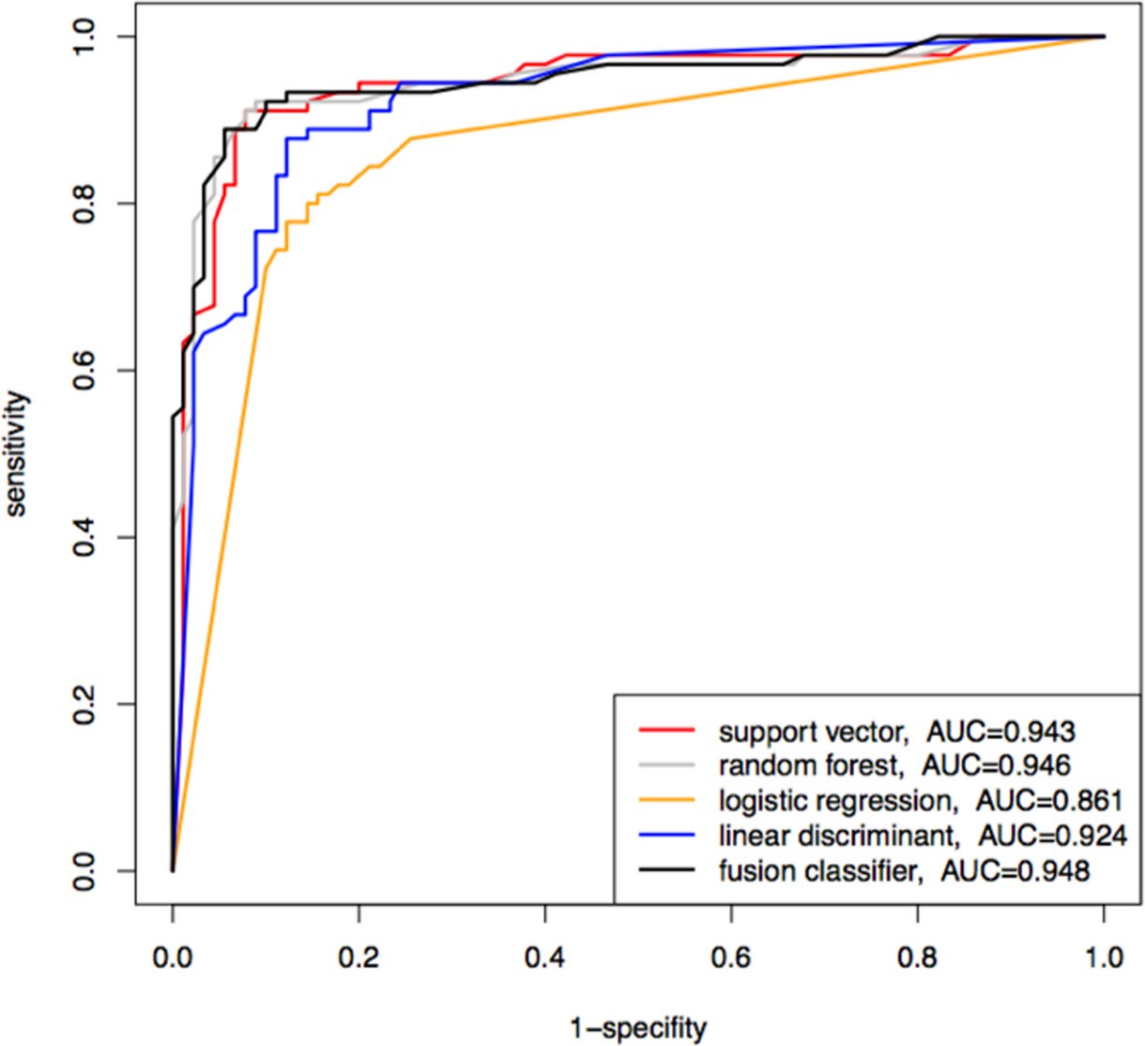
ROC curves and AUC values for RD of data set 1 (RD versus NRO). ROC curves and AUC values indicate variable diagnostic sensitivity among different classifier systems for identifying correctly classified questionnaires of patients with RD of data set 1.

For further retrospective tests, ROC curves and area under the curve (AUC) calculations were added to measure the diagnostic quality. Fig 2 shows a typical result for a fusion SVM classifier reaching AUC values of 0.948 which was outperforming each of the 4 single classifiers.

### Prospective testing

The retrospective calculations and training procedures were based on three data sets (Tab. 3) including 440 questionnaires. The remaining 715 questionnaires (1155 minus 440) were used for simulation of prospective testing. This ‘prospective’ data set included 536 ‘RD, 110 ‘NRO’, 59 ‘CD’ and 10 ‘PSY’ samples. The prospective test performs an independent evaluation for each of the three data sets. The results confirmed the sensitivity values shown in Tab. 4. Prospective testing was extended to 1155–440 = 715 questionnaires containing a bundle of different—assumed or unknown–questionnaires answered by patients. Due to the lack of confirmed diagnoses, the prospective classification results for these 715 questionnaires are not detailed in this paper.

### Diagnostic support for a potential professional user

**Fig 3:**
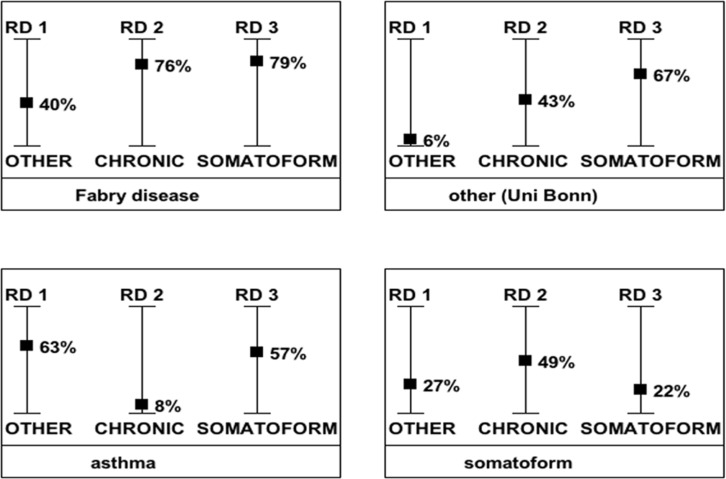
Diagnostic support for a potential professional user. Results of different patient questionnaires with a) Fabry disease (upper left), with b) an unknown diagnosis (upper right), with a c) chronic condition (below left) and with a d) somatoform disorder (below right) disease. The machine learning approach calculates these graphics, visualizing the probability values for a RD compared to other diagnoses. In a clinical setting, such a result could then be interpreted by the user in the context of the patient history.

The machine learning approach evaluates three independent data sets and therefore it delivers three probability pairs, one for each data set for RD versus NRO, for RD versus CD and for RD versus PSY. Fig 3 visualizes these ‘probability pairs’ referring to the probabilities of the RD 1, RD 2 and RD 3 disease groups. In four different examples of questionnaires, referring to a patient with Fabry disease (upper left), to a patient with inconclusive symptoms with no definite final diagnosis (NRO, upper right), to a patient with a diagnosis of asthma (lower left) and to a patient with a somatoform disorder (lower right) the results of the diagnostic suggestion for a professional user is displayed. In the Fabry patient, the relatively high probability values (40%, 76%, 79%) indicate a 79% likelihood of a RD of group 3 (including several neurological RD, such as CIDP, cluster headache, Ménière disease and Fabry disease). The results for the patient with a NRO (Fig 3, upper right) show comparably low results for RD, but indicates a 94% (100%-6% = 94%) probability for a non-rare disease (NRO). The results of the questionnaire for the asthma patient exclude a RD as well, but the probability value of 8% for the RD2 points with 92% to the alternative diagnosis of a ‘chronic disease’. The somatoform disorder is detected with 78% (100%-22% = 78%) probability and none of the three probability values for any of the RD1, RD2 and RD3 reaches more than 50% (Fig 3).

## Discussion

The main finding of this study is that patients share similar experiences during their pre-diagnostic journey despite being affected by very different RD. We utilized these experiences to create a questionnaire-based diagnostic support tool. This tool, which combines different classifier systems, effectively differentiates between answer patterns among individuals with different rare and non-rare diseases. Such a system could function as alarm for the GP to consider RD.

Diagnostic support is desirable in many different RD [19–22]. As RD are highly heterogeneous, the affected patients present with a wide variety of symptoms. However, we hypothesized that there exist a set of consistent and shared phenomena among all individuals affected by (different) RD during the time before diagnosis is established. Therefore, we aimed to identify these commonalities and developed a diagnostic support tool.

Based on our previously performed Delphi-survey we conducted interviews with individuals with different RD and designed a unique questionnaire which reflected the pre-diagnostic experiences of different individuals during their odyssey. This approach proved successful in previous projects in developing new diagnostic support tools [14,15]. The final questionnaire developed in this study contained 53 questions that were systematically distilled from interviews using the technique suggested by Colaizzi [17]. Likewise, different subjective perspectives were grouped in categories so that the final questionnaire reflected a breadth experiences.

Based on three independent data sets, the ten-fold stratified cross-validation method for the answer-pattern recognition resulted in sensitivity values of 88.9% to detect the answer pattern of a RD, 86.6% for NRO, 87.7% for CD and 84.2% for PSY.

Collectively, our data illustrates that despite suffering from different RD, patients share surprisingly similar pre-diagnosis experiences. These commonalities were qualitatively explored and successfully used to develop a questionnaire. Mathematical algorithms learned to distinguish different answer-patterns.

In our study, 183 questionnaires were answered by individuals with neurological or neuromuscular diseases. In neuromuscular diseases, diagnostic delay is common, as illustrated by a recent study in Scotland on patients with oculopharyngeal muscular dystrophy [20]. Here, symptoms were apparent for up to 20 years before the diagnosis was made. Reasons for the delay varied, and included patient denial, unspecific symptoms, and the rarity of the disease itself, but the role of the GP as gatekeeper for individuals with undiagnosed RD is eminent [20,21]. Rarity, clinical variability at presentation and lack of time for the patient history hamper rapid diagnosis in individuals with RD [22–25]. New systems to remind medical gatekeepers of rare diseases are urgently needed, as underlined by multiple reports addressing different disease groups [26,27].

Computer-aided diagnostic support goes back to the 1980s [28,29]. Using databases and statistical algorithms, scientists hoped to enhance diagnostic accuracy and reduce diagnostic mistakes [30]. Despite some successes, in the everyday life of doctors and patients, diagnoses are overwhelmingly still made exclusively by the practitioner and are usually not computer-supported. On the other hand, new digital and social media offer new opportunities to facilitate the diagnostic journey. Addressing the need for timely diagnosis, a Dutch group developed a mobile application (App) for early diagnosis of treatable diseases resulting in psychomotor delay [31]. Such examples illustrate the benefits of today’s technology, which is increasing and improving quickly, as illustrated by recent publication on diagnostic support tools for RD [32,33]. Importantly, most decision support tools (ADA Dx, FindZebra, Phenomizer) use leading symptoms for diagnosis, whereas the tool under discussion here uses the patients’ view in his/her language by using a questionnaire.

A diagnostic support tool like ours could help enhance awareness for RD. A different approach is the implementation of screening programs or targeted screening for selected RD, e.g. for alpha-1-antitrypsin deficiency in COPD patients [34]. For individuals with acromegaly, the framework of a screening program in Latin America was described by Danilowicz et al. [35]. In acromegaly patients, the delay in diagnosis is common and results in increased morbidity and mortality, whereas timely treatment would improve health and quality of life. Of note, this study included an interview with an individual with acromegaly and 12 questionnaires were answered by patients suffering from acromegaly.

During this project 12 patients with Fabry disease completed the questionnaire, and our diagnostic tool learned to detect the ‘Fabry answer pattern’ and subsequently provided correct diagnostic suggestions (Fig 3). In females suffering from Fabry disease, a delay in diagnosis results in major organ morbidity [36]. According to data from the Fabry registry, the median age at first symptoms was 13 years, but the median age at diagnosis was 31 years. Tragically, twenty percent of patients experienced major health setbacks associated with Fabry disease, partially due to the long diagnostic latency period [36]. A questionnaire-based alarm system, hinting towards the possibility of a RD, would be an easily implementable method for individuals searching for an explanation for their symptoms as well as for GPs trying to diagnose complex cases.

The hardships RD patients endure have been widely reported [37,38]. Therefore, new approaches using pattern recognition to discern which patients are suffering from ‘common’ ailments and which might have an RD are urgently needed.

Our study has several limitations. First, we performed interviews within a small and heterogeneous population. This may have resulted in a selection bias of the chosen questions. Although this may be consistent with the everyday reality of a GP who cannot ask all relevant questions due to time constraints, it also reflects the limitations of a questionnaire-based diagnostic support tool. And some questions originate from the German health system (e.g. questions 7 and 15, S1 File) and might not be 100% transferable to any other system. The diagnostic odyssey, however, is very international. And so is the patients’ impression that the health system does not help properly to find the diagnosis. A second limitation of the system is its potential biased towards detecting a RD much more accurately than, for example, a simple migraine. However, this issue could be mitigated by prospective testing and the detection of an index patient, although this is challenging in the setting of RD.

Furthermore, the training data set of 1155 questionnaires was somewhat small and by definition did not reflect all possible disease manifestations or all possible RD. In addition, certain diagnoses are more heavily represented in the data set due to particularly well-organized patient advocacy groups.

And, the set of data from patients with psychosomatic disorders is still quite small, which will need to be addressed in a future study. However, as a proof of concept, our data show that it is possible to provide a diagnostic hint by the computer-based analysis of answer patterns, which might be valuable in pre-selecting for RD patients.

Finally, the patients answered the questionnaire after knowing their diagnosis which might cause a bias. The current system was not yet systematically tested under ‘real life’ conditions, where the results are expected to be inferior.

In conclusion, our study provides evidence that a simple questionnaire and the analysis of answer patterns by machine learning technologies can result in high diagnostic accuracy in a data set of patients with different RD. Modern mathematical procedures are able to distinguish answer patterns by sifting through large amounts of data. These results give room for hope that such technologies might serve as adjunctive tool for physicians and scientists. In the future and after further testing and more prospectively collected data, pattern recognition might help to shorten the diagnostic delay even in the notoriously challenging area of RD. The value of patient observations during the pre-diagnostic time is underlined by our data. Certainly, diagnosis remains in the hands of physicians, but raising awareness for RD and easing the path to eventual diagnosis can be triggered by the tool presented here.

## Supporting information

S1 File 53-item questionnaire (German version). (PDF)

S2 File 53-Item questionnaire (English version). (PDF)

S3 File Additional information on classifier performance. (PDF)

S4 File Collection of answers (Part 1). (XLSX)

S5 File Collection of answers (Part 2). (XLSX)

S6 File Collection of answers (Part 3). (XLSX)

## Abbreviations

App: application
CD: common chronic diseases
EDS: Ehlers-Danlos syndrome
NRO: other common non-rare diseases
OTC: ornithine transcarbamylase
PSY: psychosomatic or somatoform diseases
RD: rare disease
