# Supplementary material for: Common pre-diagnostic features in individuals with different rare diseases represent a key for diagnostic support with computerized pattern recognition?
Source: PLoS One. 2019 Oct 10;14(10):e0222637. doi: 10.1371/journal.pone.0222637 (PMC6786570; doi:10.1371/journal.pone.0222637)
Supplement: S2 File — (PDF) [file pone.0222637.s002.pdf]

This questionnaire was designed to support individuals who search for a proper medical diagnosis. Some persons might think they have a rare disease, others might not believe in the diagnosis they received.

Again others might be searching for a long time because they “feel” that they are different but nobody find’s the answer for their phenomena.

These questions origin from interviews with affected individuals. Please try to answer every question – there is no wrong or right.

Your answer pattern will help your health professional to assist you finding YOUR diagnosis!

II.SEARCHING FOR REASONS

1 Did you suspect – if so since longer – that something is ‚wrong‘ with you?

2 Is it true that your complaints / irritating phenomena did not result in any reaction when you went to the doctor?

3 Did you ever had abnormal test results (e.g. ECG, blood-exam, skin) that were not further evaluated?

4 Is it difficult for you to put your complaints / irritating phenomena into words?

5 Have you ever thought that environmental factors (nutrition, living, journeys, etc) might have contributed to your complaints / irritating phenomena?

6 Have you been submitted to plenty of investigations without conclusive results?

7 Is it always for the same complaints / irritating phenomena that you consult a doctor?

8 Do you have a variety of complaints / irritating phenomena?

9 Do you remember – in the context of your complaints – a situation that was particularly threatening?

10 Do you use tricks and dodges to master restrictions during your daily life?

11 Do you avoid willfully activities that make your complaints / irritating phenomena obvious?

12. Did you initiate research regarding your complaints / irritating phenomena?

13. Would you say that your complaints / irritating phenomena were named differently (received different diagnoses) during the time?

14. Did you – whilst searching for a diagnosis – look for experts by yourself?

15. Did you ever ask for investigations from your physician?

16. Do you have the impression that your complaints/symptoms/phenomena are not taken seriously by the physician(s)?

17. Did you lose confidence into your physician(s)?

18. Did you ever reach the point to give up the search for a diagnosis?

19. Is it true that a psychological or psychosomatic disease was suspected?

20. Did a certain experience or 'light-bulb-moment' brought home the deterioration of your complaints / irritating phenomena?

III. SIGNS OF THE DISEASE

21. Do you frequently suffer from fever / elevated temperature?

22. Do you suffer from multiple complaints at the same time (e.g. cough and loss of weight / drop of performance and visual disturbance?

23. Are you constantly tired?

24. Did you observe an irritating peculiarity (e.g. strange colour of the skin; swelling) of your body?

25 Do you suffer from recurrent severe pain?

26. Do you have a noticiable drop of performance (e.g. during sport activities, climbing stairs)?

27. Did you notice pulmonary problems (shortness of breath during exercise, severe cough, breathing pauses)?

28. Did you notice gait disturbances (e.g. stumbling, tumbling, staggering)?

29. Have you been desperate due to your complaints?

30. Is it true that you have been approached by people from your environment (family, acquaintances, friends, colleagues, etc.) on your physical abnormalities?

IV. BRINGING COMPLAINTS UNDER CONTROL

31. Did you learn over the time to better assess your symptoms

32. Have you tried on your own to alleviate your symptoms (for example, by herbal medications, ointments, sports, special diet, etc.)?

33 Did you ever insist on receiving a certain treatment talking to your doctor?

V. BEING SPECIAL:

34. Is it true that you can do certain things that others can't – and vice versa?

35. Does comparing yourself with others make your complaints / irritating phenomena obvious?

36 Is it true that you have always been weaker than others (e.g. in sports)?

37. Are you frequently in the spotlight because of your complaints / irritating phenomena?

38. Do you feel ashamed because of your complaints / irritating phenomena?

39. Are your complaints / irritating phenomena occult or invisible from the outside?

40. Do you feel that others have to show consideration for you?

41. Is it true that you push back your complaints as much as possible in an effort to lead a normal life?

42. Would you say that the ambiguity about the cause of your complaints / irritating phenomena was the worst?

43. Did you ever consider that your complaints / irritating phenomena are an illusion?

VI. SOCIAL ENVIRONMENT

44. Would you say that your environment shows only little understanding for your situation?

45. Do you withhold information about your complaints from your environment (e.g. family, friends, colleagues)?

46. Is it true that you prefer staying at home (instead of going out/clubbing) since your complaints are obvious?

47. Do you have a person of trust in whom you have 100% confidence in the context of your complaints / irritating phenomena?

VII.: DAILY LIFE

48. Do you refrain from certain activities that you normally enjoy?

49. Did you change your daily routine due to your complaints / irritating phenomena?

50 Do you plan adventures (e.g. go clubbing, journeys, holidays) plan anticipatory and detailed?

51. Do you use aids / devices to get along better in the daily routine?

52. Do you consider changing your professional situation (or did you already realize a change) because of your health?

53. Is it true that – compared to others – you need more efforts to reach your goals?
