# Supplementary material for: Common pre-diagnostic features in individuals with different rare diseases represent a key for diagnostic support with computerized pattern recognition?
Source: PLoS One. 2019 Oct 10;14(10):e0222637. doi: 10.1371/journal.pone.0222637 (PMC6786570; doi:10.1371/journal.pone.0222637)
Supplement: S3 File — (PDF) [file pone.0222637.s003.pdf]

Supplementary File 3

The classifiers support vector machine (svm), random forests (rf), logistic regression (lr), linear discriminant analysis (ld) and the fusion classifier are compared on the basis of the cross-validation runs. This is done separately for each of the three data sets. For each data set, a table containing the mean rate of correctly classified questionnaires, the corresponding standard deviation and a 95% confidence interval for the rate of correctly classified questionnaires (Table S2.1, S2.2 and S2.3 for data set 1, 2 and 3, respectively).

Table S2.1 Statistics for cross-validation on data set 1.

	svm	rf	lr	ld	fusion
Mean	0,86666667	0,86666667	0,78888889	0,74444444	0,87777778
SD	0,16396995	0,11475506	0,13302433	0,12883353	0,16932044
CI.left	0,74936963	0,78457584	0,69372901	0,65228249	0,75665323
CI.right	0,9839637	0,94875749	0,88404876	0,8366064	0,99890232

Table S2.2 Statistics for cross-validation on data set 2.

	svm	rf	lr	ld	fusion
Mean	0,88888889	0,92222222	0,8	0,86666667	0,9
SD	0,09072184	0,09147473	0,11475506	0,11475506	0,12227833
CI.left	0,82399039	0,85678514	0,71790917	0,78457584	0,81252735
CI.right	0,95378739	0,9876593	0,88209083	0,94875749	0,98747265

Table S2.3 Statistics for cross-validation on data set 3.

	svm	rf	lr	ld	fusion
Mean	0,75	0,85	0,6625	0,5125	0,85
SD	0,23570226	0,12909944	0,20454896	0,26647128	0,12909944
CI.left	0,58138876	0,75764782	0,51617449	0,32187793	0,75764782
CI.right	0,91861124	0,94235218	0,80882551	0,70312207	0,94235218

The Wilcoxon-Mann-Whitney test with correction for ties was also applied to compare the different classifiers concerning their 10 rates of correctly classified questionnaires in the cross-validation runs. Tables S2.4, S2.5 and S2.6 provide the p-values for data set 1, 2 and 3, respectively.

Table S2.4 p-values of the Wilcoxon-Mann-Whitney test for the comparison of the classifier based on cross-validation on data set 1.

	svm	rf	lr	ld	fusion
svm	1	0,78090021	0,11095715	0,01336898	0,82291238
rf	0,78090021	1	0,17734742	0,0213146	0,54291065
lr	0,11095715	0,17734742	1	0,49773756	0,08535582
ld	0,01336898	0,0213146	0,49773756	1	0,01565308
fusion	0,82291238	0,54291065	0,08535582	0,01565308	1

Table S2.5 p-values of the Wilcoxon-Mann-Whitney test for the comparison of the classifier based on cross-validation on data set 2.

	svm	rf	lr	ld	fusion
svm	1	0,50457901	0,11088138	0,81034445	0,7364524
rf	0,50457901	1	0,02793955	0,32726407	0,87951677
lr	0,11088138	0,02793955	1	0,28369309	0,07816796
ld	0,81034445	0,32726407	0,28369309	1	0,54095131
fusion	0,7364524	0,87951677	0,07816796	0,54095131	1

Table S2.6 p-values of the Wilcoxon-Mann-Whitney test for the comparison of the classifier based on cross-validation on data set 3.

	svm	rf	lr	ld	fusion
svm	1	0,48737795	0,32836823	0,04238022	0,48737795
rf	0,48737795	1	0,04072398	0,00227327	1
lr	0,32836823	0,04072398	1	0,25651129	0,04072398
ld	0,04238022	0,00227327	0,25651129	1	0,00227327
fusion	0,48737795	1	0,04072398	0,00227327	1

The table show that the fusion classifier shows the best performance. However, the confidence intervals and the hypothesis test indicate that in most cases it cannot be rules out that the slightly better performance might be a matter of chance.
